# Understanding Pediatric Norovirus Epidemiology: A Decade of Study among Ghanaian Children

**DOI:** 10.3390/v12111321

**Published:** 2020-11-18

**Authors:** Belinda L. Lartey, Osbourne Quaye, Susan A. Damanka, Chantal A. Agbemabiese, Joseph Armachie, Francis E. Dennis, Christabel Enweronu-Laryea, George E. Armah

**Affiliations:** 1 West African Center for Cell Biology of Infectious Pathogens (WACCBIP), Department of Biochemistry, Cell and Molecular Biology, University of Ghana, Accra 00233, Ghana; blartey@noguchi.ug.edu.gh; 2 Department of Electron Microscopy and Histopathology, Noguchi Memorial Institute for Medical Research, College of Health Sciences, University of Ghana, Accra 00233, Ghana; sdamanka@noguchi.ug.edu.gh (S.A.D.); cagbemabiese@noguchi.ug.edu.gh (C.A.A.); armachiejoseph@gmail.com (J.A.); FDennis@noguchi.ug.edu.gh (F.E.D.); 3 Department of Child Health, University of Ghana Medical School, Korle-Bu Teaching Hospital, University of Ghana, Accra 00233, Ghana; chikalaryea@yahoo.com

**Keywords:** norovirus, gastroenteritis, onestep RT-PCR, genogroups, genotypes, Ghana

## Abstract

Understanding the epidemiology of human norovirus infection in children within Ghana and the entire sub-Saharan African region, where future norovirus vaccines would have the greatest impact, is essential. We analyzed 1337 diarrheic stool samples collected from children <5 years from January 2008 to December 2017 and found 485 (36.2%) shedding the virus. GII.4 (54.1%), GII.3 (7.7%), GII.6 (5.3%), GII.17 (4.7%), and GII.5 (4.7%) were the most common norovirus genotypes. Although norovirus GII.4 remained the predominant capsid genotype throughout the study period, an increase in GII.6 and GII.3 capsid genotypes was observed in 2013 and 2014, respectively. The severity of clinical illness in children infected with GII.4 norovirus strains was similar to illness caused by non-GII.4 strains. Since the epidemiology of norovirus changes rapidly, establishment of systematic surveillance within sentinel sites across the country would enhance the monitoring of circulating norovirus strains and allow continuous understanding of norovirus infection in Ghana.

## 1. Introduction

Globally acute gastroenteritis (AGE), the most common presentation of diarrhea disease remains one of the infectious diseases associated with a majority of morbidity and mortality [1]. After birth asphyxia, pneumonia, congenital anomalies, and neonatal sepsis, diarrhea disease remains the fifth most important cause of death in children less than five years of age [2]. Most of these deaths occur in countries within sub-Saharan Africa and Southeast Asia where there is poor sanitation and relatively poor access to safe drinking water and healthcare systems compared to other parts of the world [1]. Diarrheal deaths are rarely seen in developed countries, although those countries still record a high number of cases and hospital visitations due to the illness [3]. As a result, the diarrheal disease burden in terms of morbidity, health care use, and expenses on disease management is quite high in the developed countries compared to developing countries [4]. Until recently, rotavirus gastroenteritis was one of the most common reasons for pediatric hospitalizations, nonetheless in countries where the rotavirus vaccines have been adopted for routine use in their expanded immunization programs, the magnitude of rotavirus-associated diarrhea admissions and deaths have reduced greatly [5,6]. Vaccine effectiveness data from Ghana suggest that rotavirus-related morbidity has been reduced by ~60% [7,8]. It has been suggested that, if the remaining burden associated with other causative agents of AGE is minimized or prevented, an enormous amount of human suffering and cost to the health systems of countries could be averted [9].

Recent advances in the use of highly sensitive RT-PCR assays have made the detection of noroviruses more frequent in diarrheic samples [10]. Human noroviruses are now being recognized as an important cause of both sporadic and epidemic forms of AGE in persons of all age groups and are now considered the second leading cause of viral gastroenteritis in children less than five years of age after rotaviruses [11]. Noroviruses have been suggested to be responsible for sustaining the morbidity and mortality rates of diarrheal disease globally after the introduction of the rotavirus vaccine [12], as well as, ranking as the most common cause of gastroenteritis among pediatric populations [13,14]. Currently, noroviruses are estimated to be the cause of about one-fifth of all gastroenteritis cases in children less than five years worldwide, translating into approximately 685 million episodes of diarrhea [15] and 212,000 deaths annually [16]. Nearly 99% of these deaths occur in the middle- and low-income countries [17]. Studies by others have also shown that norovirus disease exerts a global economic burden of $4.2 billion on health systems [18].

Recent systematic reviews, using data generated from the USA, Europe, and Asia, to evaluate the role of noroviruses in AGE globally, pegged the incidence of norovirus disease at an estimated rate of 18% with a winter seasonality [19,20]. More prominently however, these reviews, as well as other reviews from less developed countries, highlighted the lack of data from the regions believed to record high numbers of mortality rates; the middle- and low-income countries [16,21]. These reviews also highlighted the need for more studies from the less endowed regions to better understand the real contribution of noroviruses to the burden of diarrheal diseases.

Current breakthroughs in the development of in vitro culture systems for human norovirus [22,23] have now made it possible for targeted antivirals, as well as potent multivalent norovirus vaccine development, with a norovirus vaccine currently advancing in Phase 2b clinical trials [24]. 

Understanding human norovirus epidemiology in children within sub-Saharan Africa, where future vaccines would have the greatest impact, is therefore essential. In this study, the prevalence, clinical features, and genotype diversity of norovirus disease over a 10-year period were investigated in children less than 5 years of age in Ghana, a country that implemented infant rotavirus vaccination in April 2012, utilizing an already established rotavirus-sentinel surveillance system.

## 2. Materials and Methods

### 2.1. Study Design and Sample Collection

Ethical and study protocols were reviewed and approved by the Scientific and Technical Committee and Institutional Review Board of the Noguchi Memorial Institute for Medical Research, University of Ghana (NMIMR-IRB 101/16-17). Samples used in this study were obtained from children under the age of five years as part of an ongoing National Rotavirus Surveillance study. Samples were obtained from the Navrongo War Memorial Hospital (WMH), Paga and Kassena East Health Centers in the Kassena Nankana District in the Northern Region of Ghana, and Agogo Presbyterian Hospital in the Ashanti region of Ghana. Other sites were the Ho Municipal and Volta Regional Hospitals in the Volta Region and the Korle-Bu Teaching and Princess Marie Louise Children’s Hospitals in the Accra Metropolitan district of the Greater Accra Region of Ghana. These study sites represent the Northern Savannah, Middle Forest, and Southern Coastal ecological zones of Ghana respectively.

### 2.2. Laboratory Analysis 

#### 2.2.1. Nucleic Acid Extraction and Norovirus RNA Amplification

Viral RNA was extracted from clarified 20% (*w/v*) fecal suspensions using the QIAamp Viral RNA Mini kit (QIAGEN, Germantown, MD, USA) according to the manufacturer’s instructions. OneStep RT-PCR was performed targeting the polymerase and capsid (P-C) overlap region of the norovirus gene using the primers MON432/GISKR and MON431/GIISKR specific for the variable regions of norovirus genogroups I and II respectively [25]. 

#### 2.2.2. DNA Purification and Nucleotide Sequencing

Based on quality and quantity of norovirus cDNA, a total of 200 positive RT-PCR products were selected for sequencing using the Sanger sequencing platform. Clean PCR products were directly purified with a MasterMix of ExoSAP-IT (Applied Biosystems, Foster City, CA, USA) or QIAquick^®^ PCR purification kit (Qiagen, Hilden, Germany) according to the manufacturer’s instructions. Norovirus positive bands were excised from gels with non-specific products and purified using the QIAquick gel extraction kit (QIAGEN, Hilden, Germany). Sequencing in both the forward and reverse directions was based on the dideoxy-chain terminator method, using the BigDye^®^ Terminator v 3.1 cycle sequencing kit (Applied Biosystems, Foster City, CA, USA) with the same norovirus genogroup specific primers as used in the previous OneStep RT-PCR procedure.

### 2.3. Data Analysis

#### Sequence Analysis and Norovirus Genotyping

Manual editing and analysis of sequences were performed by BioEdit Sequence Alignment Editor v7.2.1 program and Molecular Evolutionary Genetic Analysis (MEGA) v7.0.26 software package. Sequences from this study were compared and aligned with sequences held in the National Center for Biotechnology (NCBI) public GenBank database using the Basic Local Alignment Search Tool (BLAST) server. Genotyping results were confirmed by using an automated online genotyping tool (v.2.0) which is available on https://www.rivm.nl/mpf/typingtool/norovirus/. 

### 2.4. Statistical Analysis

All statistical analyses were performed by using STATA version 12.1 (StataCorp LP, College Station, TX, USA). Associations among variables were examined using the Chi-square test with *p*-values < 0.05 considered statistically significant. Categorical variables were reported as frequencies and percentages, and continuous variables were reported as means ± standard deviations (SDs) or as medians with interquartile ranges (IQRs). The associations between demographic data, clinical characteristics, and norovirus infections were determined by calculating odds ratios (OR) and 95% confidence intervals (CI) in logistic regression models. Norovirus seasonal variability was determined by evaluation of monthly prevalence and calendar seasons (rainy versus dry seasons). The severity of norovirus-associated acute gastroenteritis in infected children was evaluated by applying the Vesikari scoring system according to clinical manifestations [26].

## 3. Results

### 3.1. Demographic and Clinical Characteristics of Study Subjects

A total of 1337 diarrheic stool samples from children <5 year were collected from the sentinel study sites from January 2008 to December 2017 and tested for noroviruses. Enrolled study children were aged 2 weeks to 58 months with a median age of 12 months (IQR: 0.5–50 months). The proportion of children with diarrhea decreased gradually with age. The 0–24-month-old age group alone accounted for 81.0% (1077/1337) of all diarrhea cases recorded. The major clinical symptoms associated with gastroenteritis in this study were vomiting, dehydration, and fever. Vomiting was present in 71.5% (817/1139) of the cases studied whilst 57.9% (659/1139) of the children were found with mild to severe dehydration. Vomiting (39.5%; 323/817) and severe dehydration (43.2%; 64/148) were most frequently present in children between 6 and 11 months old. The mean duration of diarrhea and vomiting in study participants were 3.2 (SD: ±1.8) and 2.0 (SD: ±1.7) days, respectively. Most children (66.9%; 759/1140) reported fever with temperatures ranging between 37.1 and 38.4 °C. The demographic features associated with norovirus detection in the Ghanaian pediatric population are shown in Supplementary Table S1.

### 3.2. Norovirus Detection and Epidemiology 

**Figure 1:**
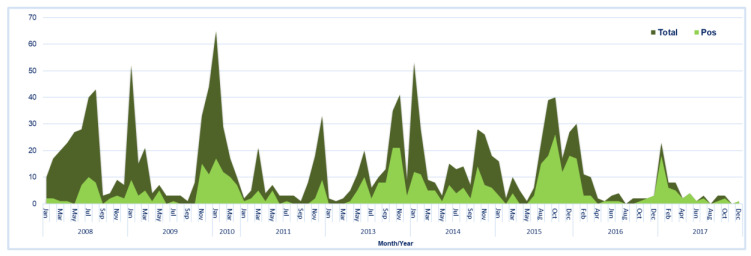
The stacked area chart represents, the total number of gastroenteritis (GE) cases tested and the number of GE cases testing norovirus-positive cases per month/year. Filled deep green area==total number of GE cases; Filled light green area==number of norovirus positive cases.

Between 2008 and 2017, norovirus was detected in 36.2% (485/1337) of the samples tested. Norovirus positivity varied significantly over the years of study (ranging from 12.5% to 75.0%; *p* = 0.000), with the highest positivity rate recorded in 2017 (Figure 1). The proportion of norovirus positive cases in the pediatric population was also observed to increase significantly from 23.0% in the pre-rotavirus vaccination years to 48.3% in the post-rotavirus vaccination years. Children had a 3.0-fold higher likelihood to be positive for norovirus infection in the post-rotavirus vaccine years when compared to the pre-vaccine years (OR 3.0; 95% CI 2.391–3.824; *p* = 0.000). The norovirus incidence in the Southern-Coastal plain was 2-fold higher (45.8% (232/506); OR 2.04; 95% CI 1.130–3.690; *p* = 0.018) compared to the Northern Savannah (30.5% (236/773); OR 1.06; 95% CI 0.590–1.903; *p* = 0.846), and Middle Forest ecological zones (29.3%; 17/58), respectively. 

Infection was not gender-dependent, however, the incidence of infection was found to be higher in males compared to females (37.9% vs. 36.7%, *p* = 0.682). Noroviruses were detected across all the age groups with a median age of infection of 13.4 months (IQR: 0.5–52 months). Infection was however common in children under two (2) years of age (Supplementary Table S1). Though not statistically significant, the peak of infection was in the 19–24 months age group (42.6%; OR 1.27; 95% CI 0.627–2.559; *p* = 0.510). A tendency to increased norovirus circulation in the late months of the year was observed, showing a seasonality trend, mostly in the post-rotavirus vaccine introduction era (Figure 1).

Of the 485 norovirus positive samples, 76.7% (*n* = 372) belonged to norovirus genogroup II (GII), 14.4% (*n* = 70) belonged to genogroup I (GI), whilst 8.9% (*n* = 43) tested positive for both GI and GII. Out of the 200 positive norovirus samples sequenced, 170 (85.0%) were successfully assigned to human norovirus capsid and/or the polymerase genotypes and four samples to other non-human calicivirus types. Approximately 71% (123/174) of the sequenced samples genotyped for both the capsid and polymerase genes, whilst 27.0% (47/174) genotyped for only the capsid region. Among the GII genogroup samples submitted for sequencing, 86.5% (152/175) were successfully assigned a capsid and/or polymerase genotype, while 72.0% (18/25) of the GI samples could also be assigned a capsid and/or polymerase genotype.

**Figure 2:**
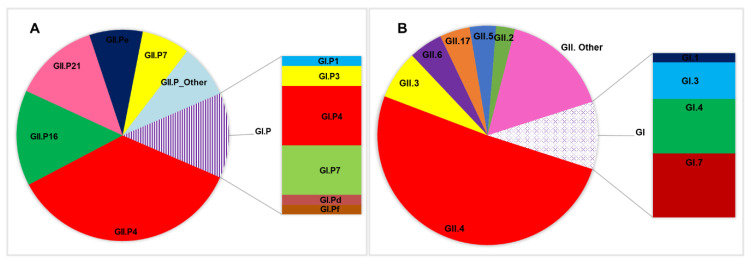
Distribution of the circulating polymerase (**A**) and capsid (**B**) genotypes in Ghanaian children between 2008 and 2017.

Of the GII genogroups, GII.[P4] was the most predominant (35.7%) polymerase genotype detected, followed by GII.[P16] (14.6%), GII.[P21] (13.0%), and GII.[P31] (8.1%). The most predominant capsid genotype detected was GII.4 (54.1%), followed by GII.3 (7.7%), GII.6 (5.3%), and GII.17 (4.7%), whilst GI.[P4] (37.5%) and GI.7 (31.3%) constituted the major detected polymerase and capsid genotypes of the GI genogroup, respectively (Figure 2). The three most common Capsid/RdRp genotype combinations were GII.4[P4] (26.5% (45/170), GII.4[P16] (9.4% (16/170), and GII.3[P21] (6.5% (11/170) [Supplementary Table S2a]. 

Results on the characterization of the norovirus genotypes and genogroups detected for each age groupings are shown in Supplementary Table S2a,b, respectively. High genotype diversity was observed in children aged 6–24 months (*n* = 17/18 capsid genotypes) compared to infected children <6 months (*n* = 10/18 capsid genotypes) and >24 months (*n* = 4/18 capsid genotypes) of age. GII.4 and GI.4 were the only norovirus capsid genotypes detected across all the different age groupings. Norovirus GII.4 capsid genotype also represented the most predominant genotype detected across all age groups with prevalence higher than 50% in each age group. On the other hand, norovirus GII.3, GII.6, and GII.17 capsid genotypes were the second, third, and fourth highest genotypes commonly detected in norovirus positive children aged between 6 and 24 months (92.3% (12/13); 88.9% (8/9); 87.5% (7/8)), respectively. All other uncommon GI and GII genotypes were sporadically detected at low prevalence in all age groups.

### 3.3. Clinical Characteristics of Norovirus Infection within the Study Population

The clinical characteristics of the children with and without norovirus infections are summarized in Table 1. While the mean diarrheal frequency (episodes/24 h period) of norovirus infected children did not differ significantly from those without norovirus infection (5.2 ± 2.5 vs. 5.5 ± 2.8, respectively, *p* = 0.05), there was a significant difference in mean diarrheal duration between the two groups (2.9 ± 1.6 vs. 3.3 ± 1.7, respectively; *p* = 0.019). Vomiting was more frequently identified in norovirus positive children compared with norovirus negative children (OR 1.56; 95% CI 1.189–2.056; *p* = 0.001). Norovirus positive children were more likely to report 2–4 vomiting episodes per day (OR 1.62; 95% CI 1.192–2.212; *p* = 0.002) and vomiting duration of up to a day (OR 1.91; 95% CI 1.282–2.847; *p* = 0.002). Norovirus infection was not significantly associated with severe dehydration (OR 1.18; 95% CI 0.798–1.735; *p* = 1.176). Norovirus infected children were however 2.7 times more likely to report with temperatures ≥ 39.0 °C (*p* = 0.002) and 4.1 times more likely to be hospitalized (*p* = 0.000). Children reporting with norovirus-associated gastroenteritis were generally more likely to be drowsy and lethargic (OR 2.71; 95% CI 1.500–4.895; *p* = 0.001) than irritable (OR 1.33; 95% CI 0.996–1.776; *p* = 0.053).

Evaluation of the severity of norovirus-associated diarrhea using the 20-point numerical score (Vesikari score) showed scores that ranged between 5–20 points. There was significant difference between the mean severity score of norovirus-infected and norovirus negative children (13.3 ± 3.0 vs. 12.5 ± 3.2, respectively; *p* = 0.005). Norovirus infection was also significantly associated with the presentation of severe gastroenteritis (V-score ≥ 11) in all the age groups of children assessed in the study (OR 1.88; 95% CI 1.393–2.529; *p* = 0.000). There was however no significant association in the clinical severity of infection in children presenting with either norovirus genogroup I (GI) or GII infection (GI: OR 0.90; 95% CI 0.324–2.506; *p* = 0.840 vs. GII: OR 0.94; 95% CI 0.401–2.237; *p* = 0.902, respectively) [Table 2].

### 3.4. Norovirus Genotype and Associated Disease Severity

Increased frequency of diarrhea (≥6 stool episodes per day) and vomiting (≥5 vomiting episodes per 24 hours) was observed in more than half of the children infected with norovirus GII.3 and GII.4 strains. Approximately 50% of children infected with norovirus GII.4 strain showed prolonged vomiting duration of more than three (3) days. Many children infected with GII.3 norovirus strain (~50%) experienced severe dehydration, whereas children infected with GII.4 and GII.17 strains experienced mild to moderate dehydration. Children infected with strains from the GI genogroup generally presented with less severe clinical symptoms, though this was not significant. 

Evaluation of the association of the clinical outcome in infected children with circulating predominant norovirus genotypes showed all patients infected with either norovirus GII.3, GII.17, or GII.21 strains presented with severe gastroenteritis (Vesikari scores ≥ 11). Moderate to severe disease (7 ≤ score ≥ 11) was observed in children infected with either GII.4, GII.6, or GII.5 strains (Table 2). Among the GI norovirus strains, all children infected with GI.3 exhibited severe diarrheal disease (Vesikari scores ≥ 11), whilst patients infected with GI.4 and GI.7 strains experienced moderate to severe diarrhea (Vesikari scores 7 ≤ score ≥ 11) [Table 2]. 

## 4. Discussion

Noroviruses are increasingly being recognized as one of the more important viral agents of childhood diarrheal disease worldwide [14,16]. To account for the high numbers of diarrheal cases still seen within our region even after the rotavirus vaccine introduction, this current study investigated the role played by noroviruses in the high burden of diarrhea disease in hospitalized Ghanaian children. This study spanned a period of ten years from 2008 to 2017 and included eight (8) sentinel sites from four different geographic locations within the country. The findings from this study showed the importance of norovirus in children under 5 years hospitalized with acute gastroenteritis post-rotavirus vaccine era and identified children ≤2 years to be the most vulnerable to a host of norovirus strains. 

Earlier studies in Ghana detected noroviruses in 10–16.5% of hospitalized children less than five years [27,28,29]. Previous studies from few countries within sub-Saharan Africa also identified noroviruses at prevalence rates ranging from 4.6% to 32.4% [30] with an average rate of 13.5% [31]. Similarly, a global systematic review and meta-analysis studies on the global prevalence of norovirus gastroenteritis conducted between 2008 and 2014 estimated norovirus to be responsible for 18% (95% CI 17–20) of all acute gastroenteritis cases in children <5 years [19]. The detection rate of 36.2% of noroviruses in the children in this study was clearly higher than those previously reported. This discrepancy might be due to the varying study periods, varying study durations, as well as the geographical distribution of the studied population. In addition, government policy on routine rotavirus vaccine administration led to a very rapid uptake of the rotavirus vaccines, leading to a decline in rotavirus associated AGE [8] and a subsequent increase in norovirus associated gastroenteritis as observed.

Though norovirus-associated diarrheal cases were seen across all the different age groups in this study, it was observed more frequently in the children between the ages of 6–24 months (66%) when compared to those below 6 months of age (16%) and above 24 months of age (9.5%). These findings are consistent with studies from reports by Shioda [32] and from other parts of Africa including Libya, Nigeria, and Madagascar that have also reported the high detection of noroviruses in stools of children between the ages of 6–24 months with gastroenteritis [33,34,35]. Children within the ages of 6–24 months have usually lost maternal antibodies and possess limited capacity to mount an effective immunological response to infection. Other contributory factors to the observed high prevalence of norovirus infection in this age group include; licking contaminated fingers and fomites during teething. This observation supports and emphasizes the suggestion that global norovirus immunization schedules (when they become available) should be completed before 6 months of age as that would have the potential of saving about 85% of the pediatric population from severe infection [36].

Unlike the temperate northern hemisphere where norovirus infections peak during the winter, the seasonality of infection on the African continent is less obvious [31,37]. In this study, norovirus seasonality in Ghana was fairly consistent and was detected in two seasonal peaks; a major peak occurring during the cool dry months of October to January and a minor peak in the wet months of May to July. Ghana has two diarrheal seasons, the first occurring during the rainy season of June to August and the second during the dry cool months of October to February, with the latter being associated with rotavirus infection [38]. The peaks of norovirus infections coincided with these two diarrheal seasonal peaks. The observations of this study were also comparable with a study by Armah and colleagues that reported the peak of norovirus detection to coincide with the cool dry months [27]. Furthermore, in other West African countries such as Burkina Faso and Nigeria, norovirus infections were also reported to have peaked in the dry season [39,40].

Of the two most prevalent human norovirus genogroups (G), GII was more commonly detected, accounting for ~80% of all positive cases tested compared to the GI’s which accounted for ~14% of cases. Even though the pathogenicity and virulence of either GI or GII norovirus strains remain speculative [41], our study showed that both genogroups were able to cause severe infections within the pediatric population; more than 80% of the cases were severe irrespective of the norovirus genogroup involved. It has been suggested elsewhere that, of the two genogroups, the GI noroviruses which are more common in environmental samples [42] tend to cause asymptomatic infections or infections with mild clinical symptoms not requiring hospitalization [43]. Since this study was from a hospital-based surveillance that enrolled children presenting with severe diarrhea, a lot of the GI cases would have been missed and hence the inappreciable GI infection rate observed. 

Noroviruses, like other RNA viruses, are naturally diverse. It was therefore not surprising that a plethora of genotypes were identified in this study; 17 polymerase and 14 capsid genotypes were identified, respectively. This diversity in genotypes is a known feature of norovirus epidemiology not only in other sub-Saharan African countries but globally [31,44]. Diversity in circulating genotypes was also observed across the different ecological regions of Ghana with the Coastal and Northern Savannah ecological zones showing the highest genotype diversity. Differences in hygiene and sanitation, as well as differences in socio-economic status across the different belts [45], could be factors driving the norovirus genotype diversity as observed. 

Overall, GII.4 (54.1%) was the most predominant genotype detected in hospitalized Ghanaian children throughout the study period. Our results are comparable with global reports in which most cases of norovirus-associated gastroenteritis were attributable to GII.4, co-circulating with other genotypes [46]. Additionally, norovirus genotypes GII.3 (7.7%) and GII.6 (5.6%) were the second and third most predominant genotypes of gastroenteritis among our study participants. Similar findings have been reported in recent systemic reviews that have tried to account for the genotypic distribution of norovirus among the pediatric population both globally [36] and within the sub-Saharan African region [16,31]. In the Coastal Savannah ecological zone, norovirus GII.5 capsid genotype was an important source of infection and was responsible for approximately 4.0% of infections post-rotavirus vaccine era. The GI capsid genotypes reported in this study are a common source of food and waterborne norovirus outbreaks [47,48] as well as their isolation from environmental specimen in several countries [42]. Their presence in clinical specimen from hospitalized children could be an indication of food or waterborne norovirus transmission considering the majority of these GI genotypes were detected from the northern belt, a rural community where good hygiene and sanitation practices are relatively poor. Environmental norovirus studies are however necessary to establish the environmental transmission route of noroviruses in this community.

Globally emerging norovirus GII.17, which has been a predominant genotype detected in recent times, particularly in the Asian countries [49,50], ranked as the fourth most common genotype, causing approximately 5% of infections in our study. Other capsid genotypes that showed relatively low prevalence included GII.1 GII.2, GII.8, GII.13, GII.21, and GI.1. The low frequency of these genotypes did not allow any analysis of their trends within our study population. 

In an attempt to evaluate the clinical implications and importance of circulating norovirus genotypes, we investigated the association between circulating norovirus genotypes and their clinical outcomes using the Vesikari severity scoring system. Infections with the GII.3 norovirus genotype were common in children <24 months and were usually associated with severe dehydration, whilst infections with GII.4 strains were associated with prolonged vomiting duration but mild to moderate dehydration. Moreover, we observed that children infected with non-GII.4 norovirus strains recorded equally severe clinical illness comparable to that caused by GII.4 norovirus strains. A probable explanation for these observations might be the naivety and minimal immune state of children to these viral strains and hence their experiencing more severe symptoms upon infection for the first time in life. Indeed, only few studies have tried to correlate norovirus genotypes to clinical features presented by infected children [51,52,53], but these studies suggested that the norovirus GII.4 genotype was more virulent compared to non-GII.4 genotypes. Shilu et al., however, reported the ability of norovirus GII.3 genotype to cause severe infections in children less than a year old [53].

## 5. Conclusions

This report describes the epidemiology of norovirus infecting genotypes in hospitalized children under five years of age with gastroenteritis in Ghana. The study confirmed the significant role played by noroviruses as a major cause of acute watery diarrhea. A detection rate of 36.2% of norovirus infection was recorded in Ghanaian children. During the 10-year surveillance period, GII.4 and GII.3 were the two most predominant norovirus strains circulating in the Ghanaian children, suggesting that any future vaccines would need to be multivalent and effective to provide protection against these two capsid genotypes and the many genotypes in circulation. Since the epidemiology of norovirus changes rapidly, establishment of systematic surveillance within sentinel sites across the country would enhance the monitoring of circulating norovirus strains and allow a continuous understanding of the state of norovirus infection in our settings. This will put us in a better position to support public health intervention strategies against childhood norovirus-associated gastroenteritis. Studies that will enhance our understanding of norovirus evolution and adaption to immunological pressures would also be critical for future vaccine effectiveness studies.

## Figures and Tables

**Table 1:** Clinical Severity of AGE according to the norovirus infection status of study participants.

	Total Tested	NoV Pos (%)	OR (95% CI)	*p*-Value
* Subject Number	1142	435	-	-
**Clinical Profile**				
**Diarrhea Duration/day**				
1–4	967	381 (39.4)	Ref	
5	73	23 (31.5)	0.71 (0.425–1.179)	0.184
≥6	102	31 (30.4)	0.67 (0.432–1.044)	0.077
**Max No. of diarrheal stools/day**				
≥6	496	166 (33.5)	Ref	
4–5	378	169 (44.7)	1.61 (1.220–2.117)	0.287
1–3	268	100 (37.3)	1.18 (0.868–1.613)	0.001
**Vomiting**				
Absent	325	100 (30.8)	Ref	
Present	817	335 (41.0)	1.56 (1.189–2.056)	0.001
**vomit Duration/day**				
0	321	99 (30.8)	Ref	
1	150	69 (46.0)	1.91 (1.282–2.847)	0.001
2	234	91 (38.9)	1.43 (1.002–2.033)	0.049
≥3	437	176 (40.3)	1.51 (1.115–2.050)	0.008
**Max No. of Vomiting episodes/day**				
0	324	103 (31.8)	Ref	
1	49	16 (32.7)	1.04 (0.548–1.975)	0.904
2–4	383	165 (43.1)	1.62 (1.192–2.212)	0.002
≥5	386	151 (39.1)	1.38 (1.010–1.880)	0.043
**Temperature (Fever)**				
37.1–38.4	759	254 (33.5)	Ref	
38.5–38.9	333	154 (46.3)	1.71 (1.315–2.225)	0.000
≥39.0	48	27 (56.3)	2.56 (1.417–4.610)	0.002
**Dehydration (as assessed by clinician)**				
None	480	151 (31.5)	Ref	
Mild	238	81 (34.0)	1.11 (0.801–1.549)	0.523
Moderate	273	150 (55.0)	2.65 (1.950–3.599)	0.000
Severe	148	52 (35.1)	1.18 (0.798–1.735)	1.176
**Treatment**				
Rehydration	216	50 (23.2)	Ref	
Hospitalization	155	86 (55.5)	4.14 (2.645–6.474)	0.000
Rehydration/Hospitalization	770	299 (38.8)	2.11 (1.488–2.984)	0.000
* Vesikari Score				
Non-Severe (<11)	273	75 (27.5)	Ref	
Severe (≥11)	864	359 (41.6)	1.88 (1.393–2.529)	0.000

* Only children with complete clinical data were included in the analysis; NoV: Norovirus; AGE: Acute gastroenteritis; Ref: Reference.

**Table 2:** Association between NoV genogroups and genotypes and clinical severity of norovirus infections in children as measured with the Vesikari scoring system.

Norovirus Type	Vesikari Score (VS)			Total
	Mild (%)	Moderate (%)	Severe (%)	
NoV Genogroup	5 (1.2)	70 (16.1)	359 (82.7)	434 (100)
GI	0	12 (18.2)	54 (81.8)	66
GII	5 (1.5)	51 (15.6)	270 (82.8)	326
GI/GII	0	7 (16.7)	35 (83.3)	42
GII Cap/Pol Genotypes	1 (0.7)	17 (11.9)	125 (87.4)	143 (100)
GII.4[P4]	0	5 (11.4)	39 (88.6)	44
GII.4[P16]	0	2 (12.5)	14 (87.5)	16
GII.3[P21]	0	0	11 (100)	11
GII.6[P7]	0	2 (28.5)	5 (71.4)	7
GII.4[P31]	0	1 (16.7)	5 (83.3)	6
GII.21[P21]	0	0	4 (100)	4
GII.17[P17]	0	0	3 (100)	3
GII.9[P7]	0	0	2 (100)	2
GII.8[P8]	0	1 (50.0)	1 (50.0)	2
GII.1[P33]	0	1 (100)	0	1
GII.2[P30]	0	0	1 (100)	1
GII.2[P31]	0	0	1 (100)	1
GII.3[P16]	0	0	1 (100)	1
GII.3[P30]	0	0	1 (100)	1
GII.4[P7]	0	1 (100)	0	1
GII.5[P16]	0	1 (100)	0	1
GII.17[P3]	0	0	1 (100)	1
GII.17[P13]	0	0	1 (100)	1
GII.4[P_nd]	0	1 (5.2)	18 (94.7)	19
GII.5[P_nd]	0	0	6 (100)	6
GII.2[P_nd]	0	2 (66.7)	1 (33.3)	3
GII.12[P_nd]	0	0	3 (100)	3
GII.6[P_nd]	0	0	2 (100)	2
GII.13[P_nd]	1 (50.0)	0	1 (50.0)	2
GII.17[P_nd]	0	0	2 (100)	2
GII.7[P_nd]	0	0	1 (100)	1
GII.10[P_nd]	0	0	1 (100)	1
GI Cap/Pol Genotype	0	3 (17.6)	14 (82.4)	17 (100)
GI.4[P4]	0	2 (40.0)	3 (60.0)	5
GI.7[P7]	0	1 (25.0)	3 (75.0)	4
GI.3[P3]	0	0	2 (100)	2
GI.1[P1]	0	0	1 (100)	1
GI.3[P13]	0	0	1 (100)	1
GI.3[P14]	0	0	1 (100)	1
GI.7[P_nd]	0	0	2 (100)	2
GI.4[P_nd]	0	0	1 (100)	1
**Total**	**1 (0.6)**	**20 (12.5)**	**139 (86.9)**	**160 (100)**

NoV: Norovirus; Mild: VS < 7; Moderate: VS = 7–10; Severe: VS ≥ 11; Cap: Capsid genotype; Pol: Polymerase genotype; [P_nd]: Polymerase genotype not determined; NB: Only children with complete clinical data were included in the VS analysis.

## Supplementary Materials

The following are available online at https://www.mdpi.com/1999-4915/12/11/1321/s1, Table S1: Demographic features associated with Norovirus detection in the Ghanaian pediatric population between 2008 and 2017, Table S2a: Age-stratified norovirus genotypes detected in children with AGE in Ghana, Table S2b: Age-stratified norovirus genogroups detected in children with AGE in Ghana.
