# Supplementary material for: Understanding Pediatric Norovirus Epidemiology: A Decade of Study among Ghanaian Children
Source: Viruses. 2020 Nov 18;12(11):1321. doi: 10.3390/v12111321 (PMC7698731; doi:10.3390/v12111321)
Supplement: Supplementary file 1 [file viruses-12-01321-s001.pdf]

viruses

Supplementary Table 1: Demographic features associated with Norovirus detection in the Ghanaian pediatric population between 2008 and 2017.

Parameters	Total Tested	NoV Pos (%)	OR (95% CI)	*p*-Value
**All Study Samples**	1337	485 (36.3)	-	-
**Study Period**				
Pre-vaccine Years (2008–2012)	652	154 (23.6)	Ref	
Post-Vaccine Years (2013–2017)	685	331 (48.3)	3.02 (2.391–3.824)	0.000
**Study Site**				
Middle Belt	58	17 (29.3)	Ref	
Southern Belt	506	232 (45.8)	2.04 (1.130–3.690)	0.018
Northern Belt	773	236 (30.5)	1.06 (0.590–1.903)	0.846
**Gender**				
Male	663	251 (37.9)	Ref	
Female	534	196 (36.7)	0.95 (0.752–1.205)	0.682
* Unknown	140	38 (27.1)	-	-
**Age (Months)**				
>36	46	17 (37.0)	Ref	
25–36	74	29 (39.2)	1.10 (0.514–2.349)	0.807
19–24	115	49 (42.6)	1.27 (0.627–2.559)	0.510
12–18	371	136 (36.7)	0.99 (0.523–1.863)	0.968
6–11	382	135 (35.3)	0.93 (0.494–1.758)	0.829
<6	209	81 (38.8)	1.08 (0.558–2.089)	0.820
* Unknown	140	38 (27.1)	-	-
**GC of Patient**				
Normal	392	115 (29.3)	Ref	
Irritable	461	164 (35.6)	1.33 (0.996–1.776)	0.053
Drowsy and Lethargic	51	27 (52.9)	2.71 (1.500–4.895)	0.001
Unconscious	1	0	-	-

* Missing data excluded from all statistical analysis; CI: Confidence Interval; OR: Odds Ratio; NoV: Norovirus; GC: General Condition of patient; Ref: Reference.

Supplementary Table 2a: Age-stratified norovirus genotypes detected in children with AGE in Ghana.

	**Age (Months)**				**Total No. of Cases**
	**<6**	**6–24**	**>24**	*** Unknown**	
No. of typed samples	34	126	7	7	174
GI Genotypes					18
Cap/Pol					14
GI.4[P4]	3	1	1	0	5
GI.7[P7]	2	2	0	0	4
GI.3[P3]	0	2	0	0	2
GI.1[P1]	1	0	0	0	1
GI.3[P13]	0	1	0	0	1
GI.3[P14]	0	1	0	0	1
Cap/Pol[nd]					4
GI.7	0	2	0	1	3
GI.4	0	1	0	0	1
GII Genotypes					152
Cap/Pol					109
GII.4[P4]	9	34	2	0	45
GII.4[P16]	3	13	0	0	16
GII.3P[21]	0	10	1	0	11
GII.4[P31]	2	4	0	2	8
GII.6[P7]	1	6	0	0	7
GII.21[P21]	0	5	0	0	5
GII.17[P17]	0	3	0	0	3
GII.9[P7]	0	1	1	0	2
GII.8[P8]	0	2	0	0	2
GII.1[P33]	0	1	0	0	1
GII.2[P30]	0	1	0	0	1
GII.2[P31]	0	1	0	0	1
GII.3[P16]	0	1	0	0	1
GII.3[P30]	0	1	0	0	1
GII.4[P7]	0	1	0	0	1
GII.5[P16]	0	1	0	0	1
GII.17[P3]	0	1	0	0	1
GII.17[P13]	0	1	0	0	1
GII.17[P31]	1	0	0	0	1
Cap/Pol[nd]					43
GII.4	4	13	2	3	22
GII.5	4	2	0	0	6
GII.12	1	2	0	1	4
GII.2	1	2	0	0	3
GII.13	1	1	0	0	2
GII.17	0	2	0	0	2
GII.6	0	2	0	0	2
GII.7	0	1	0	0	1
GII.10	0	1	0	0	1
**Total**	**33**	**123**	**7**	**7**	**170**

No. of typed samples: Number samples genotyped; * Unknown: Information on Age was not available; Cap: Capsid genotype; Pol: Polymerase genotype; P[nd]: Polymerase genotyped not determined; NB: Four (4) capsid sequences presumptively typed as other non-human calicivirus were excluded from subsequent analysis.

Supplementary Table 2b: Age-stratified norovirus genogroups detected in children with AGE in Ghana.

	**Age (Months)**				**Total N (%)**
	**< 6 N (%)**	**6–24 N (%)**	**> 24 N (%)**	*** Unknown N (%)**	
**No. Pos Samples**	**81 (16.7)**	**320 (66.0)**	**46 (9.5)**	**38 (7.8)**	**485 (100)**
**NoV Genogroups**					
GI	14 (20.6)	43 (63.2)	11 (16.2)	2 (2.9)	70 (14.4)
GII	60 (17.8)	248 (73.6)	29 (8.6)	35 (9.4)	372 (76.7)
GI/GII	7 (16.7)	29 (69.1)	6 (14.3)	1 (2.3)	43 (8.9)

No. Pos Samples: Number of positive samples; * Unknown: Information on Age was not available.
